# Ectopic pancreatic adenocarcinoma in Meckel’s diverticulum: a case report

**DOI:** 10.1186/s40792-024-01843-8

**Published:** 2024-02-23

**Authors:** Shoichi Inokuchi, Kohjiro Shirabe, Satoshi Tsutsumi, Hiroomi Takayama, Takahiro Terashi, Kazuhiro Yasuda, Masahiko Ikebe, Toshio Bandoh, Junpei Wada, Shogo Urabe, Tohru Utsunomiya

**Affiliations:** 1 Department of Surgery, Oita Prefectural Hospital, 2-8-1 Bunyou, Oita, 870-8511 Japan; 2 Department of Pathology, Oita Prefectural Hospital, Oita, Japan

**Keywords:** Chemotherapy, Ectopic pancreatic adenocarcinoma, Meckel’s diverticulum, Rare cancer, Surgery

## Abstract

**Background:**

Malignant neoplasms arising from Meckel’s diverticulum are rare and an adenocarcinoma in Meckel’s diverticulum originating from ectopic pancreatic tissue is even rarer. Herein, we report a patient with an ectopic pancreatic adenocarcinoma in Meckel’s diverticulum who was successfully treated with surgery and chemotherapy.

**Case presentation:**

A woman in her sixties presented to another hospital with abdominal pain. Plain computed tomography suggested an abdominal tumor and she was referred to our hospital. Enhanced computed tomography revealed a 23-mm low-density tumor in the abdominal cavity. Surgery was performed with a tentative diagnosis of a mesenteric tumor, such as a gastrointestinal stromal tumor, schwannoma, or lymphoma. First, we inspected the peritoneal cavity with a laparoscope. This revealed numerous nodules in the small bowel mesentery, suggesting peritoneal dissemination. A 20-mm-diameter white tumor was found in the small intestine and diagnosed as a small intestinal cancer. The small intestine was partially resected laparoscopically through a small skin incision. The patient’s postoperative course was uneventful, and she was discharged on postoperative day 9. Pathological examination revealed well-differentiated adenocarcinoma in the small intestine. The tumor had developed from a sac-like portion protruding toward the serosal side and had a glandular structure lined with flattened atypical cells. Neither pancreatic acinar cells nor islets of Langerhans were evident, suggesting a Heinrich type 3 ectopic pancreas. The final diagnosis was an adenocarcinoma originating from an ectopic pancreas in Meckel’s diverticulum. After a smooth recovery, the patient commenced chemotherapy for pancreatic cancer.

**Conclusions:**

We present a very rare case of ectopic pancreatic carcinoma in Meckel’s diverticulum.

## Background

Meckel’s diverticulum, a common congenital anomaly, is found in 0.6–2.3% of all autopsy cases [1, 2]. Meckel’s diverticulum is a true diverticulum found approximately 100 cm toward the oral side in the ileocecal region [3]. The cells lining Meckel’s diverticulum are pluripotent, and the diverticulum may contain jejunal mucosa, duodenal mucosa with Brunner’s glands, gastric mucosa, and pancreatic tissue. Although rare, these ectopic tissues may be associated with malignant degeneration [4]. Malignant neoplasms arising from Meckel’s diverticulum are uncommon, for example, the frequency of adenocarcinoma arising from Meckel’s diverticulum is approximately 12–16% [5]. Cancers originating from ectopic tissue in Meckel’s diverticulum are mostly from ectopic gastric mucosa; cancers originating from ectopic pancreatic tissue are very rare [6].

In the present report, we describe a patient with a very rare ectopic pancreatic adenocarcinoma in Meckel’s diverticulum who underwent surgery and chemotherapy.

## Case presentation

**Fig. 1:**
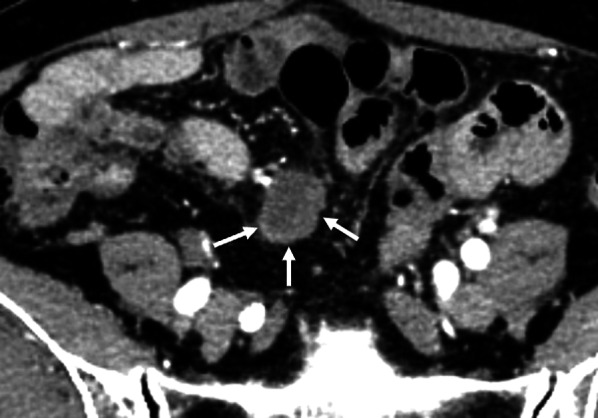
Enhanced computed tomography image showing a 23-mm low-density tumor in the abdominal cavity (white arrows)

A woman in her sixties presented to another hospital with abdominal pain. She had a history of bronchial asthma, rheumatoid arthritis, and two cesarean sections. Plain abdominal computed tomography (CT) suggested an intraperitoneal tumor, and she was referred to our hospital. She had no smoking history, and she drank alcohol only occasionally. There were no abnormal results on laboratory tests; in particular, the serum carcinoembryonic antigen concentration was 8.9 ng/mL, and the carbohydrate antigen 19-9 concentration was 1.1 ng/mL. Enhanced CT revealed a 2-mm low-density tumor in the abdominal cavity (Fig. 1). Plain pelvic magnetic resonance imaging showed a tumor with internal heterogeneity and high signal intensity in the mesentery on T2-weighted imaging, and diffusion restriction was observed. Considering these findings, our provisional diagnosis was an intra-abdominal tumor, such as a gastrointestinal stromal tumor, schwannoma, or malignant lymphoma. To confirm the diagnosis, we decided to perform laparoscopic tumor resection.

**Fig. 2:**
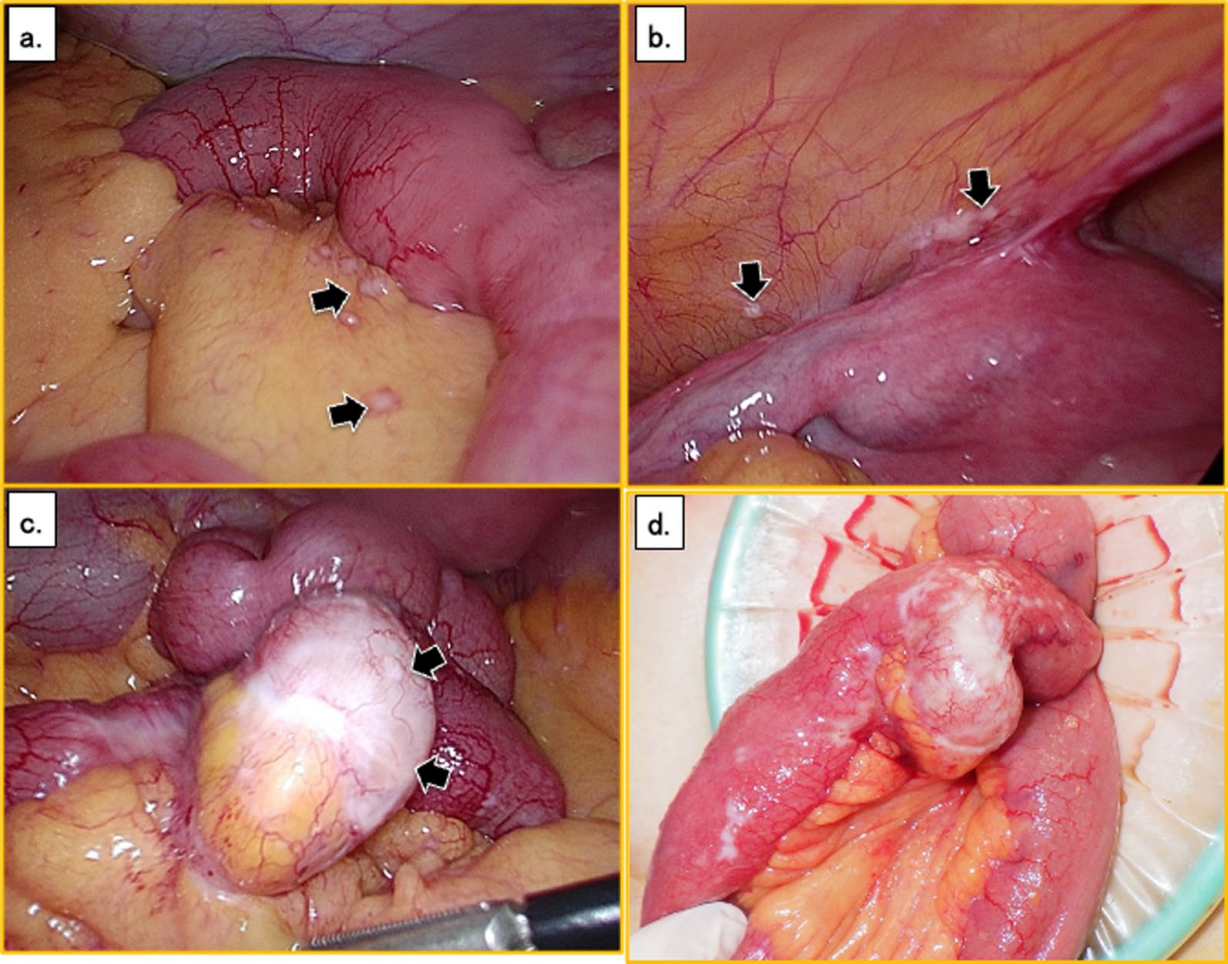
Intraoperative findings. **a** There are numerous nodules in the small intestinal mesentery (black arrows). **b** There are also disseminated nodules in the pouch of Douglas (black arrows). **c** There is a 2-cm white tumor in the small intestine (black arrows). **d** The tumor was located approximately 130 cm on the oral side, toward the ileocecal valve

First, we inspected the peritoneal cavity with a laparoscope. This revealed numerous nodules in the small bowel mesentery (Fig. 2a), and the pouch of Douglas (Fig. 2b), indicating peritoneal dissemination of the cancer. A 20-mm-diameter white tumor was found in the small intestine near the disseminated nodules (Fig. 2c), which we diagnosed this as a small intestinal cancer. We then made a small skin incision and inspected the small intestine (Fig. 2d). The tumor was located approximately 130 cm on the oral side, toward the ileocecal valve. Because of the obvious peritoneal dissemination, we performed small bowel resection, with harvesting of some lymph nodes and peritoneal nodules. The operation time was 1 h and 35 min, and the intraoperative blood loss volume was 10 mL. The patient’s postoperative course was uneventful, and she was discharged on postoperative day 9.

**Fig. 3:**
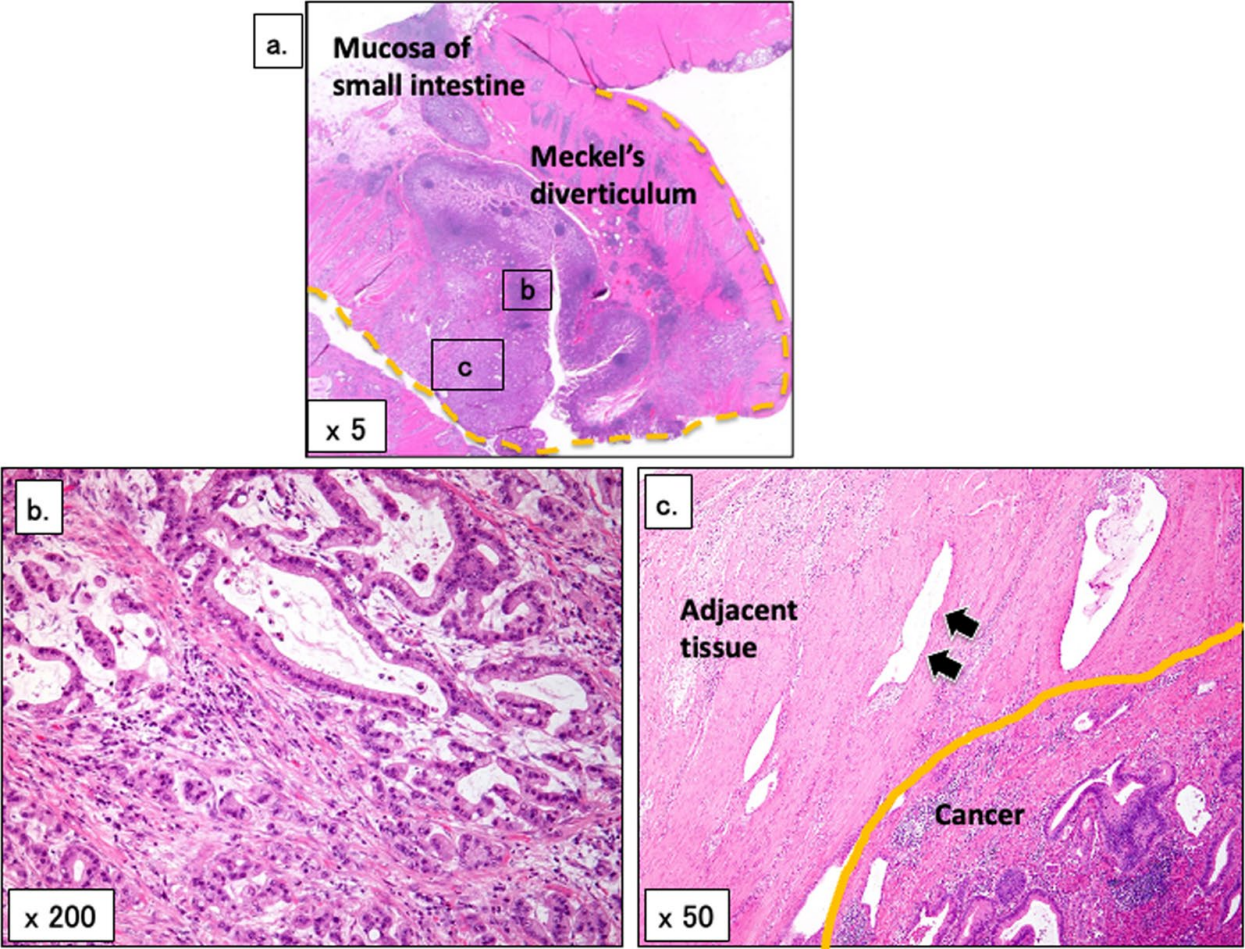
Histological findings with HE staining. **a** Photomicrograph showing a sac-like structure within the muscularis propria. The structure is a true diverticulum protruding from the small intestine (dashed yellow line). The cancerous area is shown as a Square b, and the cancer and adjacent tissue as Square c. **b** Photomicrograph showing atypical columnar epithelium that has grown in a tubular manner in the mucous membrane of the diverticulum. **c** Photomicrograph showing an enlarged space covered by flattened atypical cells in the tissue of Meckel’s diverticulum and in contact with the cancer. This tissue is suggestive of ectopic pancreas. There is no gastric mucosa, intestinal mucosa, acinar cell, or islets of Langerhans. HE, hematoxylin and eosin

**Fig. 4:**
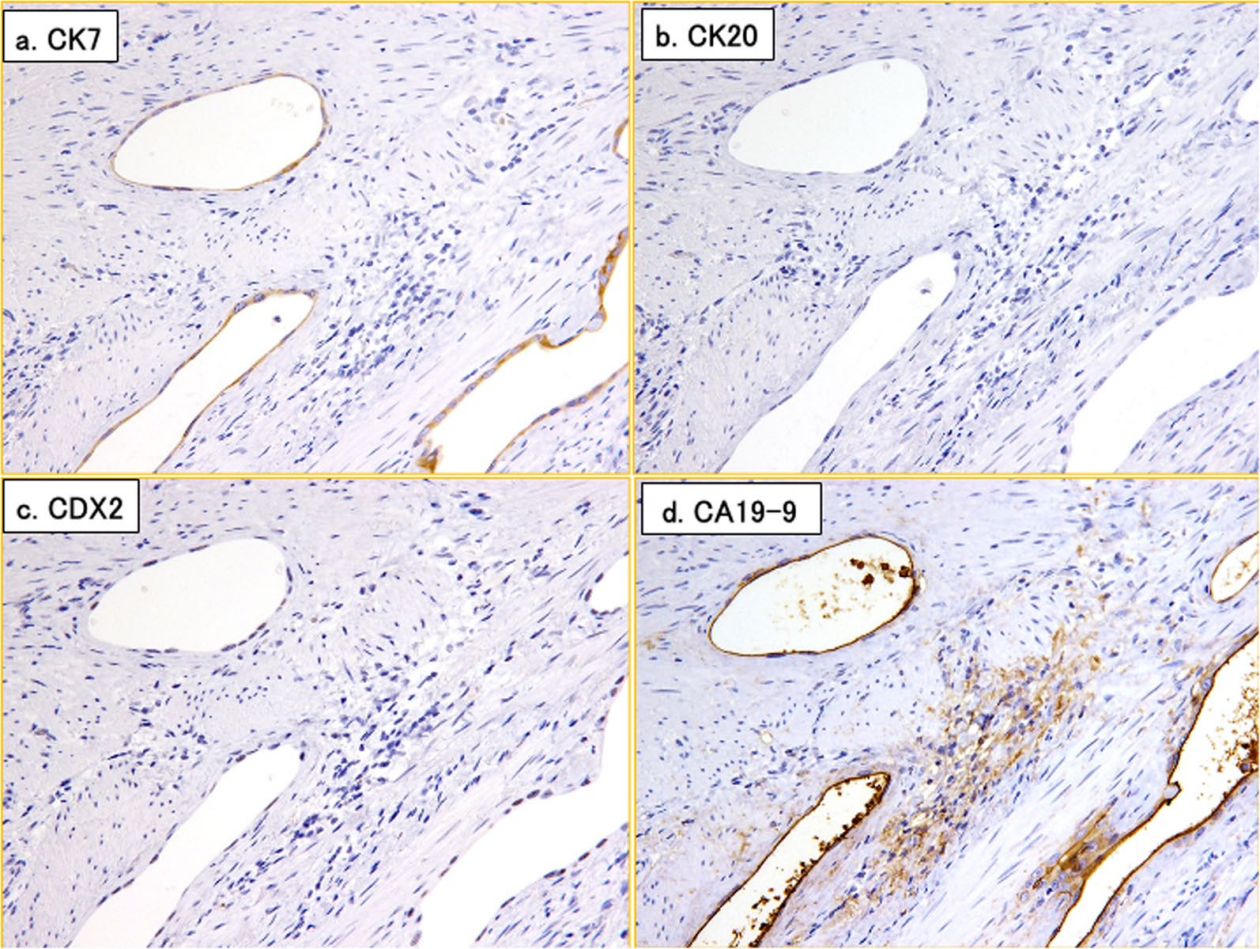
Immunohistochemical findings for the resected cancer tissue. Photomicrographs showing **a** CK7 positivity; **b** CK20 negativity; **c** CDX2 negativity; and **d** CA19-9 positivity. *CK* cytokeratin, *CDX2* *CDX2* gene, *CA19-9* carbohydrate antigen 19-9

Macroscopic examination of the resected specimen revealed a tumor protruding to the contralateral side of the mesentery. Microscopic examination revealed that the tumor was in a true diverticulum and was a well- to poorly differentiated adenocarcinoma (Fig. 3a, b). Enlarged spaces covered by flattened atypical cells were observed adjacent to the cancer (Fig. 3c). These findings suggested that the cancer was derived from ectopic mucosa. No gastric mucosa, intestinal mucosa, acinar cells, or islets of Langerhans were detected. Immunohistochemical staining of the tumor was positive for cytokeratin 7 and carbohydrate antigen 19-9, and negative for cytokeratin 20 and caudal type homeobox 2 (Fig. 4a–d). The localization of the tumor and adjacent tissue suggested that the origin of the tumor was Heinrich type 3 ectopic pancreas, which was consistent with the findings on immunohistochemical staining. Adenocarcinoma was also found in all resected lymph nodes and disseminated nodules. Thus, the final pathological diagnosis was an adenocarcinoma in Meckel’s diverticulum, pT4N2M1 (UICC 8th edition).

We treated the cancer as an unresectable pancreatic cancer, commencing gemcitabine (GEM) as a therapeutic measure 6 weeks after the surgery. Because this rare cancer arose from ectopic pancreatic tissue, we performed comprehensive genetic profiling (CGP) using Foundation One^®^ caudal type homeobox (Chugai Pharmaceutical Co., Tokyo, Japan). CGP analysis showed no actionable mutations; therefore, we added nanoparticle albumin-based paclitaxel (nab-PTX) from the third course of GEM. Follow-up CT showed that the disseminated nodules were stable, in accordance with the Response Evaluation Criteria in Solid Tumors, 12 months after surgery. Currently, 15 months after surgery, the patient continues to receive combination chemotherapy with GEM + nab-PTX and remains asymptomatic.

## Discussion

Meckel’s diverticulum was first described in 1598 and named in 1809 when Johann Friedrich Meckel described its embryological origin [7]. Meckel’s diverticulum is a vestigial remnant of the vitello-intestinal duct, which acts as a communicating tract between the embryonic yolk sac and primitive mid-gut in the first few weeks of development [8]. Although Meckel’s diverticulum is the most common malformation of the gastrointestinal tract, it occurs in only 2–4% of individuals [9, 10]. Meckel’s diverticulum is rarely symptomatic in adults [9].

Tumors in Meckel’s diverticulum are rare, comprising 0.5%–3.2% of all gastrointestinal tumors. Neuroendocrine tumors are the most common, accounting for 33–44% of Meckel’s diverticulum tumors [11], followed by leiomyosarcomas at 18–25%, adenocarcinomas at 12–16%, and gastrointestinal stromal tumors at 12% [5, 12, 13]. The chief manifestations of an adenocarcinomas in Meckel’s diverticulum are abdominal pain, abdominal masses, and intestinal obstruction. Even if such a tumor is visualized as an abdominal mass on CT imaging, localization and early diagnosis are difficult [3]. We identified an intra-abdominal tumor preoperatively in our patient. Intraoperatively, this was seen as a small intestinal tumor. Pathological examination revealed a true diverticulum with a muscular layer protruding from the small intestine in a sac-like structure, leading to the diagnosis of a Meckel’s diverticulum carcinoma.

Heterotopic pancreatic tissue is uncommon, with a reported incidence of 0.55–13.7% [14, 15]. Malignant change is even rarer [16]. Ectopic pancreatic cancer is defined as the presence of ectopic pancreatic tissue and transition to a cancer that histologically resembles pancreatic cancer. In our case, we wished to identify the origin of the cancer cells to determine the optimal chemotherapeutic regimen. It was difficult to decide whether the tumor was derived from the intestine or ectopic tissue purely based on the cell morphology and the results of immunohistochemical staining. However, there was an expanded cavity covered with markedly atypical cells in the tissue adjacent to the cancer, suggesting that the cancer had originated from ectopic tissue. Furthermore, because no gastric mucosa, intestinal mucosa, acinar cells, or islets of Langerhans were detected in the resected specimen, the tumor was considered to be derived from Heinrich type 3 ectopic pancreatic cells.

A few patients with ectopic pancreatic adenocarcinoma have been reported; however, such tumors rarely arise in Meckel’s diverticulum. To our knowledge, only one such case has been reported [3]. The patient in the previous report underwent laparotomy with a preoperative diagnosis of an intra-abdominal tumor. Similar to findings in our case, intraoperative findings revealed multiple peritoneal disseminations. The patient received no chemotherapy, and died from tumor recurrence 6 weeks after undergoing surgery [3]. Thus, we believe ours is the first report of successful treatment with surgery and chemotherapy of an ectopic pancreatic adenocarcinoma arising from Meckel’s diverticulum.

Unsurprisingly, given the small number of cases, there are no established chemotherapeutic regimens for tumors in Meckel’s diverticulum. After establishing a histopathological diagnosis, we first adopted GEM because this is the standard chemotherapy for pancreatic cancer. We also submitted the cancer specimens for CGP analysis because of the rarity of ectopic pancreatic cancer. No actionable mutation was found, and to our knowledge, this is the first report of CGP analysis of this rare type of cancer. Currently, our patient has stable disease, in accordance with the Response Evaluation Criteria in Solid Tumors and continues to receive chemotherapy (GEM + nab-PTX) 15 months after surgery.

## Conclusions

We report the first patient with ectopic pancreatic adenocarcinoma arising from Meckel’s diverticulum who was treated successfully with surgery and chemotherapy. Moreover, we confirmed the effectiveness of chemotherapeutic regimens for pancreatic cancer. Accumulation of data from CGP in this rare entity is desirable.

## Data Availability

All data generated or analyzed during this study are included in the published article.

## Abbreviations

CT: Computed tomography
GEM: Gemcitabine
CGP: Comprehensive genetic profiling
nab-PTX: Nab-paclitaxel
